# Mixed Form of Hirsutism in an Adolescent Female and Laser Therapy

**DOI:** 10.5812/ircmj.9410

**Published:** 2014-06-05

**Authors:** Besa Gacaferri Lumezi, Aferdita Goci, Violeta Lokaj, Hatixhe Latifi, Natyra Karahoda, Ganimete Minci, Drita Telaku, Antigona Gercari, Allma Kocinaj

**Affiliations:** 1 Department of Physiology and Immunology, University Clinical Center of Kosovo, Pristina, Kosovo; 2 Child and Adolescent Mental Health Center, University Clinical Center of Kosovo, Pristina, Kosovo; 3 Department of Pediatrics, University Clinical Center of Kosovo, Pristina, Kosovo; 4 Department of Dermatology, University Clinical Center of Kosovo, Pristina, Kosovo

**Keywords:** Hirsutism, PCOS, NC-CAH, Lasers

## Abstract

**Introduction:**

Hirsutism is a common disorder of excess growth of terminal hair in an androgen-dependent male distribution in women, including the chin, upper lip, breasts, back, and abdomen. It is very important to identify the etiology of hirsutism and adequate treat is prior to any cosmetic therapy.

**Case Presentation:**

The case was a 17-year-old female with severe hirsutism, oligomenorrhea, and obesity. She was evaluated to identify the etiology and diagnosed as a case of polycystic ovarian syndrome (PCOS), nonclassic congenital adrenal hyperplasia (NC-CAH), and hyperandrogenic insulin-resistant acanthosis nigricans (HAIR-AN) syndrome, which is a rare combination of hirsutism etiology. She was successfully treated according to the underlying pathology, and laser photoepilation was used as the preferred hair removal method.

**Discussion:**

Establishing the etiology, using the evidence–based strategies to improve hirsutism, and treating the underlying disorder, are essential for proper management of women with hirsutism.

## 1. Introduction

Hirsutism is a common disorder of excess growth of terminal hair in women in areas characteristic for male distribution, such as chin, upper lip, breasts, back, and abdomen (1). Classically, hirsutism is considered a marker of increased androgen levels in females with increased production of androgens (i.e. testosterone), either by the adrenals or due to an ovarian disease. Ovarian types of hyperandrogenism are polycystic ovarian syndrome (PCOS) and ovarian tumors. Adrenal types include Cushing's syndrome, androgen-producing tumors, and congenital adrenal hyperplasia (CAH), most commonly due to 21-hydroxylase deficiency. Less common causes include the hyperandrogenic insulin-resistant acanthosis nigricans syndrome (HAIR-AN) (2).

PCOS affects 4%-12% of women of reproductive age. It is a conglomeration of symptoms with varied presentations such as hirsutism, acne, alopecia, anovulatory cycles, and obesity. According to ESHRE/ASRM consensus workshop at Rotterdam in 2003, diagnosis of PCOS is based on presence of any two of the following:

1. chronic anovulation,
2. clinical/biochemical parameters for hyperandrogenism, and
3. polycystic ovaries on ultrasonography (3).

The disorder is commonly accompanied by insulin resistance and infertility. Women often initiate medical care for a cluster of PCOS symptoms (infertility, hirsutism, and irregular menstrual cycles) that ultimately are not the most concerning medical consequences of PCOS (diabetes mellitus (DM), coronary artery disease (CAD), endometrial hyperplasia/cancer) (4). CAH due to steroid 21-hydroxylase (21OH) deficiency is one of the most common inborn endocrine disorders and is inherited as an autosomal recessive disease. Molecular abnormalities of the *CYP21A2* gene coding for the steroid 21OH enzyme, lead to various degrees of impaired cortisol and aldosterone synthesis and androgen excess (5). Mild late congenital adrenal hyperplasia is much more common than the classic form. Women with mild congenital hyperplasia often present hirsutism, oligomenorrhea, or infertility.

## 2. Case Presentation

A 17-year-old female referred with growth of terminal hair in androgen sensitive areas. Hirsutism was evaluated according to the Ferriman-Gallwey score. The total value was 34. She had severe hirsutism over sideburns, upper lip, and chin. She appeared very anxious and had low self-esteem with family history of hirsutism and diabetes. mellitus. She did not have oily skin and acne. Menarha had started when she was 14 years old. Oligomenorrhea was present. She was not taking any medication. Hirsutism had appeared three years before she came for visit. Her weight was 98 kg, height 163 cm, and BMI 36.9 kg/m^2^. She had central (android) obesity, and increased waist-to-hip ratio (> 0.80). Blood pressure was 160/100 mmHg. Acanthosis nigricans was present.

Laboratory investigation was performed with radioimmunoassay (RIA) and Immunoradiometric assay (IRMA) methods, in the Laboratory of Endocrinology, Department of Physiology and Immunology, University Clinical Center of Kosovo, Prishtina, Kosovo, in January 2012. Blood was taken in the early follicular phase, (before 8:00 in the morning, and for the evening cortisol, at 16:00). Blood was taken for assessing the progesterone level in the middle of cycle (Table 1).

Oral glucose tolerance test (OGTT) was also performed. We measured the blood sugar before she drank liquid containing 75 g of glucose. We took blood to measure glucose, insulin and C-peptide every 30 and 60 minutes, for three hours (Tables 2 and 3).

Ultrasound tests of ovaries and abdomen did not show any changes, abnormal fluid, or mass. Magnetic resonance of head did not show any changes as well.

**Figure 1.**
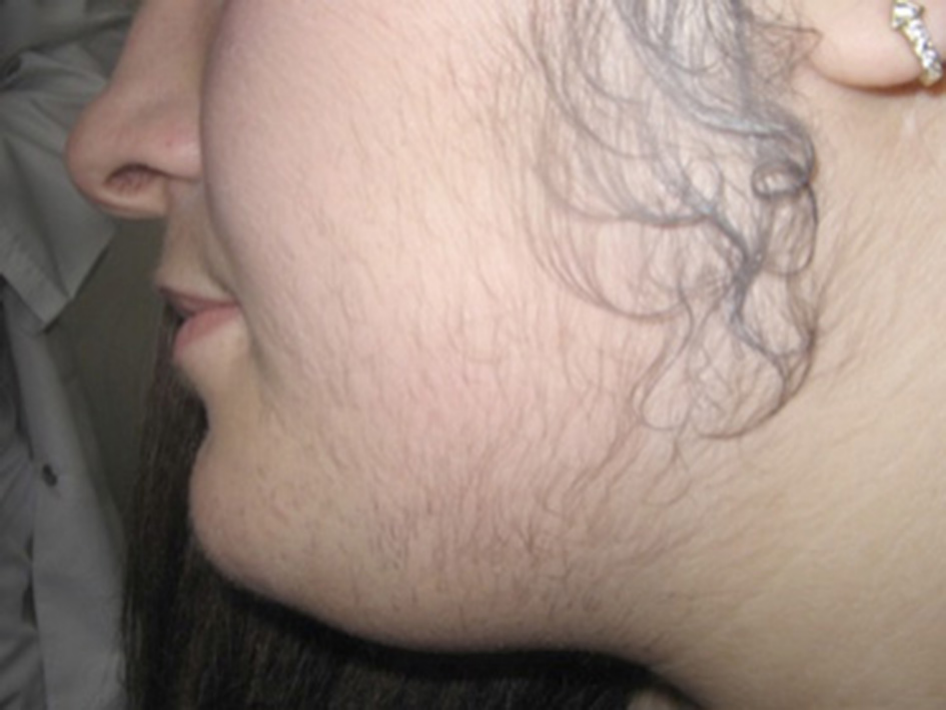
Before Therapy

**Figure 2.**
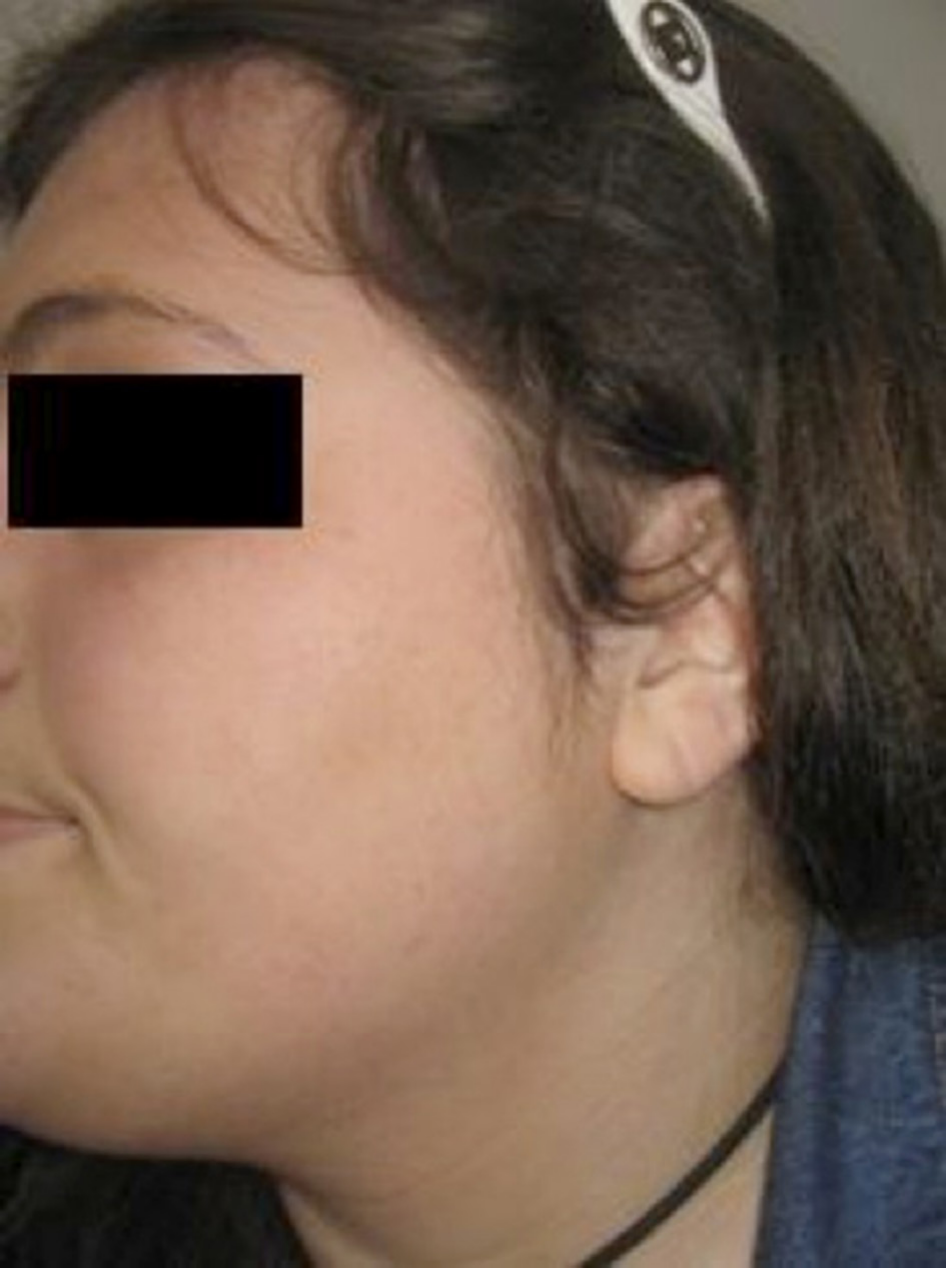
During Therapy

Etiology of her hirsutism was CAH, PCOS without polycystic ovaries but with oligomenorrhea, clinical and biochemical hyperandrogenism, insulin resistance, hypertension, obesity, and acanthosis nigricans. Metformin and cyproterone acetate/ethinyl estradiol were prescribed for her. She was treated with long-pulse alexandrite laser, 755 nm. After medical therapy and four laser treatment sessions at six-week intervals, there were marked improvements in hirsutism. Her body weight was reduced by 15 kg. Her follow up after six months showed improvements in hirsutism and regular menstrual cycle. She seemed very satisfied with the changes, her self-esteem was much higher, she reported feeling very happy (Figures 1 and 2)

**Table 1.** Levels of Hormones in the Early Follicular Phase ^a^

Hormones	Serum	Normal Range
**FSH, IU/L**	2.67	2.2-15
**LH, IU/L**	48.37	0.8-18
**Prolactin, mLU/L**	289.93	30-800
**Estradiol, pmol/L**	459.88	210-833
**Progesterone, nmol/L**	10.02	17-75
**Cortisol-morning, nmol/L**	670.23	260-720
**Cortisol-evening, nmol/L**	92.42	50-350
**TSH, nIU/mL**	6.07	0.3-5.0
**T3, nmol/L**	1.75	1.3-2.9
**T4, nmol/L**	100.99	58-166
**Total testosterone, nmol/L**	5.73	< 3
**Free testosterone, pg/mL**	5.02	0.45-3.17
**Androstenedione, nmol/L**	17.46	0.7-10.8
**DHEAS, MmL/L**	6.74	0.81-9.0
**SHBG, nM**	9.02	30-100
**17-OH- progesterone, nmol/L**	8.26	0.45-3.3
**ACTH, pg/mL**	105.97	< 40

^a^ Abbreviations: ACTH, adrenocorticotropic hormone; DHEAS, dehydroepiandrosterone sulfate; FSH, follicle-stimulating hormone; LH, luteinizing hormone; SHBG, sex hormone-binding globulin.

**Table 2.** Oral Glucose Tolerance Test of Serum ^a^

OGTT, h	0 min	30 min	60 min	120 min	180 min
**Glucose, mmol/L**	4.8	9.06	9.9	7.0	3.4
**Insulin, μIU/L**	45.4	77.4	190.1	118.9	45.2
**C-Peptide, ng/mL**	7.45	10.62	16.20	11.2	7.8

^a^ Abbreviation: OGTT, oral glucose tolerance test.

**Table 3.** Normal Ranges of Oral Glucose Tolerance Test ^a^

OGTT, h	0 min	30 min	60 min	120 min	180 min
**Glucose, mmol/L**	4.4-6.6	5.0-7.5	5.8-8.8	5.0-7.2	4.4-6.1
**Insulin, μLU/L**	5.7-12	32-80	30-58	10.4-24.8	3.8-13.2
**C-Peptide, ng/mL**	1-2.1	3.5-10.2	6.3-12.1	7.2-11.8	1.0-3.4

^a^ Abbreviation: OGTT, oral glucose tolerance test.

## 3. Discussion

PCOS is a heterogeneous disorder, in which patients can present hyperandrogenism, menstrual irregularities including oligomenorrhea, amenorrhea and infertility, and dysmetabolic syndrome that includes impaired glucose tolerance, hyperlipidemia, and obesity (6). Hyperpigmentation or acanthosis nigricans may also be observed in 5% of obese women with insulin resistance. Ovary is usually source of excess androgen in women with PCOS. Hyperinsulinemia further exacerbates the problem by stimulating the thecal cells and reducing the sex hormone-binding globulin (SHBG). Insulin resistance can occur in up to 50% of patients with PCOS.

Increase in weight also results in reduction of SHBG, thereby increasing the free testosterone. Therefore, weight loss is an extremely important measure in treatment of hirsutism for women with PCOS. To be diagnosed with PCOS by Rotterdam criteria, a women must have two of the following three manifestations: irregular or absent ovulation, elevated levels of androgenic hormones, and/or enlarged ovaries containing at least 12 follicles each (7).

Of adolescent females, 25% have multifollicular ovaries, and polycystic-type ovaries can occur in up to 20%–30% of reproductive-age females and 10% of healthy, regularly menstruating ones, making the differentiation of “normal” versus “abnormal” ovaries difficult for even experienced specialists. Moreover, transvaginal or transabdominal ultrasound is often inappropriate for pediatric patients, particularly virginal girls (8). This was the reason of not performing transvaginal ultrasound for our patient.

Nonclassic congenital adrenal hyperplasia (NC-CAH) due to P450c21 (21-hydroxylase) deficiency is a common autosomal recessive disorder due to mutations in the *CYP21A2* gene. NC-CAH can be present in childhood, adolescence, and adulthood. Symptoms include hirsutism, acne, alopecia, anovulation, and menstrual dysfunction. In a multicenter study, the most common symptoms among adolescent and adult women were hirsutism (59%), oligomenorrhea (54%), and acne (33%) (9). NC-CAH is diagnosed by confirming 17-hydroxyprogesteron and androstenedione (10).

Treatment of hirsutism should be undertaken using combination therapy, including androgen suppression, peripheral androgen blockade, mechanical/cosmetic amelioration, and destruction of the unwanted hairs. The most popular treatments for hirsutism are oral contraceptive (OC) medications, which suppress circulating luteinizing hormone (LH) and follicle-stimulating hormone (FSH), leading to a decrease in ovarian androgen production. They may also decrease adrenal androgen production by a mechanism not yet clear. The progestin in the birth control pill can lead to antagonism of 5α-reductase and androgen receptor (11). Metformin treatment significantly improves insulin sensitivity in insulin-resistant patients (12). Hirsutism can cause a lot of psychological problems. Affected women may have anxiety and depression. In one study on hirsute women suffering from PCOS, the average time spent per week dealing with facial hair was 104 minutes and majority of women reported checking facial hair frequently in mirrors and by touch, and 75% showed clinically significant anxiety (13). Laser-assisted hair removal is a well-tolerated and effective technique for patients who desire permanent reduction of hair growth. The ideal patient for laser hair removal is light skin with black course hair. Establishing the etiology, using the evidence-based strategies to improve hirsutism, and treating the underlying disorder are essential for proper management of women with hirsutism.
